# Differences in oral health status between cancer patients: a case–control observational study

**DOI:** 10.1186/s12903-025-06963-7

**Published:** 2025-12-01

**Authors:** C. Rupe, G. Gioco, M. Tranfa, F. Scilla, A. Schiavelli, F. Cairo, J. Galli, G. Scambia, C. Rengo, C. Lajolo

**Affiliations:** 1 Head and Neck Department, Fondazione Policlinico Universitario A. Gemelli—IRCCS, School of Dentistry, Università Cattolica del Sacro Cuore, Largo A. Gemelli, 8, Rome, 00168 Italy; 2 Department of Clinical and Experimental Medicine, Research Unit in Periodontology and Periodontal Medicine, University of Florence, Florence, Italy; 3 Head and Neck Department, Fondazione Policlinico Universitario A. Gemelli—IRCCS, Institute of Otolaryngology, Università Cattolica del Sacro Cuore, Largo A. Gemelli, 8, Rome, 00168 Italy; 4 Division of Gynecologic Oncology, Fondazione Policlinico Universitario A. Gemelli - IRCCS, Università Cattolica del Sacro Cuore, Largo A. Gemelli, 8, Rome, 00168 Italy; 5 Department of Neurosciences, Reproductive and Odontostomatological Sciences, University of Naples “Federico II”, Naples, 80138 Italy

**Keywords:** Oral health, Head and neck cancer, Bone metastatic cancer, Antiresorptive drugs, Radiotherapy, Oral assessment, Dental evaluation, Risk factors, Smoking, Preventive dentistry

## Abstract

**Objectives:**

The aim of this observational case‒control study was to compare the oral health (OH) status of Bone metastatic cancer (BMC) patients (case Group 1), head and neck cancer HNC patients (case Group 2) and systemically healthy patients (control group). The secondary objective was to identify any risk factors associated with poor OH status.

**Methods:**

This study was conducted from 2018–2024, and all patients were evaluated at the Oral Medicine, Head and Neck Department—Fondazione Policlinico Universitario A. Gemelli—IRCSS, Rome. The OH status was clinically and radiographically evaluated using the DMFT index, a full periodontal chart and a radiological examination (orthopantomography). The OH status was defined as “poor” in patients with stage III or IV periodontitis and/or a DMFT score ≥ 13. The study protocol was approved by the Ethics Committee of the Università Cattolica del Sacro Cuore (ID-5746). Univariate statistical analysis was performed to detect associations between different clinical variables and OH. The associated variables were subjected to multivariate logistic regression to retrieve the independent risk factors for poor OH.

**Results:**

A total of 510 (170 per group) patients were analyzed. Logistic regression analysis revealed that HNC patients showed significantly worse oral conditions when compared to BMC patients and controls. HNC patients showed an OR of 2.36 (95% CI: 1.35–4.13, *p* = 0.003) for poor OH when compared to control group. No differences were found between BMC patients and control group. Smoking habits (OR: 3.22, 95% CI: 1.93–5.35, *p* < 0.0001), and age > 70 years (OR: 17.44, 95% CI: 8.78–34.66, *p* < 0.0001) were other significant risk factors for poor OH. The risk decreased for younger patients: for patients aged 60–69 years, the OR was 5.17 (95% CI: 2.74–9.75, *p* < 0.0001); for patients aged 50–59 years, it was 4.22 (95% CI: 2.30–7.76, *p* < 0.0001).

**Conclusions:**

HNC patients exhibit significantly poorer OH than BMC patients and healthy controls, highlighting the need for enhanced dental care in oncologic management. Nevertheless, smoking habits and age remain important risk factors.

**Supplementary Information:**

The online version contains supplementary material available at 10.1186/s12903-025-06963-7.

## Background

Improvements in the survival rate of cancer patients have recently increased the interest of clinical practitioners and researchers on oral care management to prevent any predictable oral complications [1].

In particular, dental professionals are mostly involved in the management of patients suffering from head and neck cancer (HNC) and bone metastatic cancer (BMC) due to the well-known plethora of adverse events caused by anti-neoplastic therapies [2–4].

For HNC, a multidisciplinary approach involving different specialists, such as oral oncologists, has been shown to improve the overall survival rate of patients [5, 6]. A recent cross-sectional study revealed that the oral health (OH) of HNC patients is often compromised even before the beginning of cancer treatment. In that cohort, 146/195 patients (74.9%) had poor OH [7]. A recent prospective cohort study revealed that 78.3% of HNC patients require at least one tooth extraction (TE) before the beginning of radiotherapy (RT), for a mean of 3.9 TEs for each patient [8]. Furthermore, although the efforts of dental professionals are primarily aimed at the prevention of osteoradionecrosis (ORN) [9, 10], other dental and oral conditions have been reported in HNC patients, such as dental caries [11–13], oral mucositis [14] hyposalivation [15] and tooth necrosis [16], highlighting the need for comprehensive dental management of HNC patients.

Bone metastatic cancer (BMC) patients also need thorough and accurate dental management, especially considering the risk of developing medication-related osteonecrosis of the jaws (MRONJ) when antiresorptive (AR) drugs are prescribed. Furthermore, the application of AR drugs has increased in oncologic patients, given their widespread prescription for Cancer Treatment Induced Bone Loss (CTIBL) [17] and malignant hypercalcemia [18]. Poor OH has been considered a risk factor for MRONJ [19], and dental diseases should be carefully treated and prevented, especially considering improvements in the life expectancy of these patients.

Thus, BMC and HNC patients require specific dental care to prevent and manage the adverse effects of RT and AR therapy, but an epidemiological and comparative study assessing the oral health of these patients would be desirable to increase the awareness of professionals (both dentists and oncologists) about the oral conditions of their patients.

The OH conditions of BMC patients have been scarcely investigated, probably due to lack of correlation between oral health and primary cancer. Nevertheless, the topic has become interesting considered the side effects of AR treatments. Considering the absence of anatomical correlations between the oral cavity and BMC and the different risk factors for HNC and BMC, the OH conditions of these two cohorts might significantly differ. However, direct comparisons between these two cohorts of patients are currently lacking and might be desirable, to highlight similarities of differences among the two cohorts, with potential implications for the most appropriate preventive strategies for each patient.

The aim of this observational case‒control study was to compare the OH status between BMC patients (case Group 1) and HNC patients (case Group 2) at the time of dental evaluation before scheduled oncologic treatments compared with a control group of systemically healthy subjects at an outpatient university hospital. Moreover, the secondary goal was to identify any risk factors associated with poor OH status.

## Materials and methods

### Study design

This study is a retrospective case‒control study. All patients were previously evaluated at the Oral Medicine, Head and Neck Department—Fondazione Policlinico Universitario A. Gemelli—IRCSS, Rome, between 2018 and 2024.

Two prospective cohort studies, which aimed to evaluate the OH of HNC and BMC patients, were approved by the Ethics Committee of the Università Cattolica del Sacro Cuore (ID-2132 and ID-4139) and were registered at ClinicalTrials.gov (ID: NCT04009161, registration date: 19/06/2019 and ID: NCT06457776, registration date: 08/06/2024). The present study originated from a retrospective analysis of the clinical charts of the patients enrolled in the two studies, and it was approved by the Ethics Committee of the Università Cattolica del Sacro Cuore (ID-5746). Five hundred and ten (510) patients were enrolled in the study and matched by age.

Experimental procedures were conducted according to the principles of the Declaration of Helsinki on study involving human subjects, as revised in 2004.

### Participants

- Case Group 1 included patients affected by BMC who received a dental evaluation prior to the administration of antiresorptive drugs.
- Case Group 2 included patients affected by HNC who received a dental evaluation prior to RT.

The control group included systemically healthy patients seeking a dental evaluation at the Dental Department, Head and Neck Department—Fondazione Policlinico Universitario A. Gemelli—IRCSS.

### Inclusion criteria

- Case Group 1: BMC patients or patients suffering from CTIBL who received a prescription of antiresorptive drugs and were aged > 18 years.
- Case Group 2: patients with an HNC diagnosis and an indication for RT who were aged > 18 years.
- Control group: systemically healthy patients (ASA I and II) aged > 18 years.

### Exclusion criteria

- Case Group 1: patients who had already received RT in the head and neck region or had oral metastasis and patients treated by means of major oncological surgery involving the oral cavity.
- Case Group 2: patients who had already received RT in the head and neck region, patients who previously received AR drugs. Patients for whom it was impossible to accurately evaluate OH conditions: in particular, patients were not included when the outcomes of oncologic surgery were incompatible with dental procedures to diagnose caries and periodontitis (inability to retract the tongue or the buccal mucosa and/or maximum mouth opening < 10 mm).
- Control group: patients with previous oncological treatments, systemic diseases or medical treatments known to have a significant effect on oral health (i.e., diabetes, Sjögren’s syndrome).

### Data sources and measurements

All patients received a dental evaluation before RT or AR therapy, with the support of an orthopantomograph (OPT). Anamnestic and demographic data were recorded, with a specific focus on exposure to risk factors for dental and oncologic diseases. A clinical evaluation of the following parameters was subsequently performed: complete periodontal charting and dental caries diagnosis. The methods applied for the diagnostic assessment have already been reported in detail in a paper reporting the OH conditions of the HNC patient cohort [7]. Briefly, the diagnosis of caries was performed through a clinical examination with the help of a dental explorer and a mouth mirror—and when in doubt, with the support of an intraoral radiograph (periapical or bitewing)—performed with the help of film holders (Dentsply Sirona, Rome, IT). The DMFT score was then recorded.

Clinical evaluation of periodontitis was performed according to international standards by an experienced periodontist (L.C.) using an NCP15 periodontal probe. After data collection, the periodontal cases were staged according to the 2017 classification.

The number of teeth lost due to periodontitis, as well as the M parameter (teeth missed due to caries), were evaluated by analyzing previous radiographic examinations provided by the patients. When previous images were not available, the patients were asked about the reason for their previous TE.

Although the treatment plan was different among the 3 groups, because of the different needs given the different overall clinical substrates, each case was evaluated for this study with the same parameters defining OH. Then, the subjects who needed dental therapies but were not receiving RT or AR therapy were treated more conservatively, which is beyond the scope of what is reported in this article.

According to this clinical evaluation, some teeth were considered to be in need of extraction (TE) when they met one of the following conditions:

- Third molars suffering from pericoronitis [20]
- Periapical lesions [21]
- Caries extending toward the bony crest (nonrestorable) [22]

Severe periodontal damage, defined as follows:

- Hopeless teeth: Teeth where attachment loss approximates the apex of the root circumferentially, combined with a high degree of tooth hypermobility (type III) [23].
- Teeth with an “unfavorable” prognosis: Teeth affected by uncontrollable factors [24], including probing pocket depth (PPD) ≥ 7 mm, clinical attachment loss (CAL) ≥ 50% of the root, tooth mobility grade ≥ 2, and furcation involvement grade ≥ 2 [25].

Notably, among the control group, many of these conditions did not actually lead to an immediate TE, while more conservative treatment plans were established. Nevertheless, they have been categorized as above to allow statistical comparability.

### Endpoints

The differences in OH among the cohorts were set as the primary outcome. OH was defined as a dichotomous variable, defined by the DMFT score and periodontal diagnosis. OH wasclassified as “poor” in patients with stage III or IV periodontitis and/or a DMFT score ≥ 13 and “good” only in patients with lower values of the two parameters. The reasons beyond these choices are described in more detail in a previous cross-sectional study [7]: briefly, the DMFt score of 13 has been reported to be the mean value of DMFt in non-developing countries. Stage III and IV periodontitis define “severe” periodontitis, according to the 2017 classification.

### Sample size

The present case–control study included a total of 170 patients per group, matched for age in a 1:1 ratio, thus resulting in a total of 510 patients. This sample was chosen to achieve at least a power of 90% with a 99% two-sided confidence level, an odds ratio (OR) of 2.45, and a prevalence of exposure (i.e., the presence of poor OH) in the HNC group of 75% [7, 26]. The calculation was performed according to the Fleiss method with continuity correction with the software Epi Info V7 (Center for Disease Control, Atlanta, USA).

### Statistical analysis

The clinical and demographic characteristics of the sample were described by means of descriptive statistics. Qualitative variables are described as absolute and percentage frequencies. The normality of the quantitative variables was assessed by the Shapiro‒Wilk test, and the variables were summarized as the mean and standard deviation (SD), if normally distributed, or as the median and interquartile range (IQR), otherwise.

Differences between groups for quantitative variables were assessed by the ANOVA test in the case of normally distributed variables, whereas the Kruskal‒Wallis test was used in the case of a nonnormal distribution. Differences in qualitative variables were assessed by the χ^2^ test or Fisher’s exact test, as appropriate.

The variables associated with poor OH in the univariate analysis were entered into a multivariate logistic regression model by inserting the cohort of patients as covariates and OH as the outcome variable. Logistic regression analysis was performed in order to identify independent risk factors for poor OH. The same analyses were performed for the outcomes of a DMFT score ≥ 13 and severe periodontitis. Linear regression models were used to investigate the factors influencing the outcome of TE. Stepwise backward analyses were performed using significant variables in the bivariate analyses. The level of significance (*p* > 0.05) was considered an exclusion criterion for stepwise backward analysis. A P value < 0.05 was considered statistically significant. All the statistical analyses were performed using the IBM SPSS Statistics software (IBM Corp, IBM SPSS Statistics for Apple, Version 25.0, Released 2017, Armonk, NY).

## Results

**Fig. 1:**
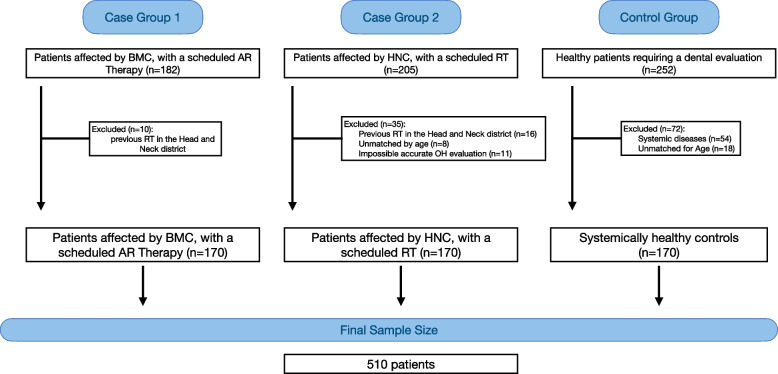
STROBE Flow-chart of the studies. A final sample of 510 was obtained. The figure also describes the reasons for exclusion of 10 patients in BMC group, 35 patients in HNC group and 72 patients in control group

Five hundred and ten (510) patients were ultimately included in the analysis. A flowchart of the study is illustrated in Fig. 1.

The final sample consisted of 170 patients per group. The mean age was 60.33 years (SD: 13.46, min–max: 22–92). The demographic data are shown in Table 1, as well as the results of the univariate analysis comparing the distribution of these characteristics among the three groups. The distribution of smokers among the three groups significantly differed: only 21/170 BMC patients were smokers (12.4%), whereas 109 (64.1%) HNC patients and 47 controls (27.7%) were smokers (χ^2^ test *p* < 0.0001). Similarly, the sex of the included patients was significantly different: BMC patients were mainly females (142/170, 83.4%), whereas in the other two groups, the percentage of females was lower: 65/170 (38.2%) in the HNC group and 58/170 (34.1%) in the control group (χ^2^ test *p* < 0.0001). No differences were found between groups for age, since patients were matched by age (*p* = 0.426). Only four patients were heavy drinkers, all of whom were in the HNC cohort.

**Table 1:** Baseline characteristics of the included patients, stratified according to the studied population

**Variable**		**HNC**	**BMC**	**Controls**	**Total**	**Statistical significance**
Sex	Men	105 (61.8%)	28 (16.6%)	112 (65.9%)	245 (48%)	
	Women	65 (38.2%)	142 (83.4%)	58 (34.1%)	265 (52%)	**χ2 Test —*p* < 0.0001**
	Total	170 (100%)	170 (100%)	170 (100%)	510 (100%)	
Age (years)	Mean (Range, SD)	59.9 (22–92; SD: 13.2)	61.4 (34–90; SD: 12.6)	59.6 (25–90; SD: 14.5)	61.0 (22–92; SD: 13.3)	ANOVA Test –* p* = 0.426
Age (decades)	0–49	35 (20.6%)	33 (19.4%)	35 (20.6%)	103 (20.2%)	
	50–59	50 (29.4%)	49 (28.8%)	50 (29.4%)	149 (29.2%)	
	60–69	40 (23.5%)	40 (23.5%)	40 (23.5%)	120 (23.5%)	
	> 70	45 (26.5%)	48 (28.3%)	45 (26.5%)	138 (27.1%)	χ2 Test —*p* = 0.91
	Total	170 (100%)	170 (100%)	170 (100%)	510 (100%)	
Tumor Site	Breast	0 (-)	129 (75.9%)	0 (-)	129 (25.3%)	
	Prostate	0 (-)	18 (10.6%)	0 (-)	18 (3.5%)	
	Oral cavity	41 (24.1%)	0 (-)	0 (-)	41 (8.1%)	
	Oropharynx	39 (22.9%)	0 (-)	0 (-)	39 (7.7%)	
	Rhinopharynx	20 (11.8%)	0 (-)	0 (-)	20 (3.9%)	
	Salivary Glands	14 (8.3%)	0 (-)	0 (-)	14 (2.8%)	
	Larynx	40 (23.5%)	0 (-)	0 (-)	40 (7.8%)	
	Other	16 (9.4%)	23 (13.5%)	0 (-)	39 (7.6%)	
	No Cancer	0 (-)	0 (-)	170 (100%)	170 (33.3%)	-
	Total	170 (100%)	170 (100%)	170 (100%)	510 (100%)	
Smoking	Smokers	109 (64.1%)	21 (12.4%)	47 (27.7%)	177 (34.7%)	
	Non smokers	61 (35.9%)	149 (87.6%)	123 (72.3%)	333 (65.3%)	**χ2 Test —*p* < 0.0001**
	Total	170 (100%)	170 (100%)	170 (100%)	510 (100%)	
Diabetes	Yes	7 (4.1%)	13 (7.6%)	0 (-)	20 (3.9%)	
	No	163 (95.9%)	157 (92.4%)	170 (100%)	490 (96.1%)	**χ2 Test —*p* < 0.0001**
	Total	170 (100%)	170 (100%)	170 (100%)	510 (100%)	

SD Standard deviation

Table 2 reports the distributions of the outcome variables within the three populations and the results of the corresponding univariate analysis. Statistically significant differences were found between groups, for the following variables: DMFT, Periodontitis (stage and grade), TEs, OH. In particular, the HNC population presented the highest prevalence of periodontitis, higher mean DMFT scores, more teeth to be extracted, and a greater prevalence of poor OH than both the BMC and control groups did. In particular, the prevalence of severe periodontitis was 54% (92/170) in the HNC group, 18.3% (31/170) in the BMC group, and 24.1% (41/170) in the control group (χ^2^ test *p* < 0.0001). The prevalence of caries followed a similar pattern: HNC patients had a mean DMFT score of 16.9 (SD: 9.2), the BMC group had a score of 9.3 (SD: 6.9), and the control group had a score of 12.4 (SD: 7.5).

**Table 2:** Distribution of the outcome variables, according to the oncologic population

**Variable**		**HNC**	**BMC**	**Controls**	**Total**	**Statistical significance**
DMFt ≥ 13	Yes	109 (64.1%)	48 (28.2%)	73 (42.9%)	230 (45.1%)	
	No	61 (35.9%)	122 (71.8%)	97 (57.1%)	280 (54.9%)	**χ2 Test—*p*** < 0.0001
	Total	170 (100%)	170 (100%)	170 (100%)	510 (100%)	
DMFt	Mean (Range, SD)	16.9 (0–32; SD: 9.2)	9.3 (0–32; SD: 6.9)	12.4 (0–32; SD: 7.5)	12.9 (0–32; SD: 8.6)	**ANOVA Test –** ***p*** < 0.0001
Periodontitis (Stage)	No Periodontitis	37 (21.8%)	77 (45.3%)	66 (38.8%)	180 (35.3%)	
	Stage 1	20 (11.8%)	31 (18.2%)	37 (21.8%)	88 (17.2%)	
	Stage 2	21 (12.4%)	31 (18.2%)	26 (15.3%)	78 (15.3%)	
	Stage 3	35 (20.6%)	17 (10%)	27 (15.8%)	79 (15.5%)	
	Stage 4	57 (33.4%)	14 (8.3%)	14 (8.3%)	85 (16.7%)	**χ2 Test —*p*** = 0.001
	Total	170 (100%)	170 (100%)	170 (100%)	510 (100%)	
Periodontitis (Grade)	No Periodontitis	37 (21.8%)	77 (45.3%)	66 (38.8%)	180 (35.3%)	
	Grade A	38 (22.4%)	42 (24.7%)	54 (31.8%)	134 (26.3%)	
	Grade B	56 (32.9%)	29 (17.1%)	27 (15.9%)	112 (21.9%)	
	Grade C	39 (22.9%)	22 (12.9%)	23 (13.5%)	84 (16.5%)	**χ2 Test —*p*** = 0.001
	Total	170 (100%)	170 (100%)	170 (100%)	510 (100%)	
Edentulism	Yes	8 (4.8%)	4 (2.4%)	0 (-)	12 (2.4%)	
	No	162 (95.2%)	166 (97.6%)	170 (100%)	498 (97.6%)	χ2 Test —*p* = 0.1
	Total	170 (100%)	170 (100%)	170 (100%)	510 (100%)	
Teeth to be extracted	Mean (Range, SD)	2.7 (0–15; SD: 3.5)	0.9 (0–9; SD: 1.8)	1.5 (0–20; SD: 2.4)	1.7 (0–20; SD: 2.8)	**ANOVA Test –** ***p*** = 0.003
Oral Health	Poor	132 (77.6%)	76 (44.7%)	95 (55.9%)	303 (59.4%)	
	Good	38 (22.4%)	94 (55.3%)	75 (44.1%)	207 (40.6%)	**χ2 Test —*p*** < 0.0001
	Total	170 (100%)	170 (100%)	170 (100%)	510 (100%)	

SD Standard deviation

Furthermore, no statistically significant differences were found in OH outcomes among different HNC populations or AR drug populations (i.e., different tumor sites or different drug dosages).

Furthermore, the following clinical characteristics were associated with the outcomes of interest (i.e., poor OH, DMFT score, severe periodontitis): smoking habit, sex, oncological cohort, and age.

Table 3 shows the results of the logistic regression for DMFT score, severe periodontitis and OH and of the linear regression analysis for TE. When the above-mentioned significant variables were put in the multivariate logistic regression analysis, the following risk factors remained significant as predictors for poor OH: smoking habit, HNC and age, while sex lost its significance.

**Table 3:** Results of logistic regression analysis for DMFt, Severe Periodontitis and OH and of the linear regression analysis for TE

Poor OH			
Predictors	OR	95% CI	*p*-value
Oncologic cohort			
Control	1.0 (Ref)		
BMC	0.81	0.47–1.38	0.430
HNC	2.36	1.35–4.13	**0.003**
Age (decades)			
0–49	1.0 (Ref)		
50–59	4.22	2.30–7.76	** < 0.0001**
60–69	5.17	2.74–9.75	** < 0.0001**
>70	17.44	8.78–34.66	** < 0.0001**
Smoking			
No	1.0 (Ref)		
Yes	3.22	1.93–5.35	** < 0.0001**
Sex			
Female	1.0 (Ref)		
Male	1.62	0.97–2.61	0.06

DMFt ≥ 13			
	OR	95% CI	*p*-value
Oncologic cohort			
Control	1.0 (Ref)		
BMC	0.51	0.29–0.87	**0.013**
HNC	2.00	1.19–3.35	**0.008**
Age (decades)			
0–49	1.0 (Ref)		
50–59	5.4	2.76–10.57	** < 0.0001**
60–69	4.96	2.48–9.92	** < 0.0001**
>70	16.36	8.07–33.13	** < 0.0001**
Smoking			
No	1.0 (Ref)		
Yes	2.72	1.70–4.36	** < 0.0001**
Sex			
Female	1.0 (Ref)		
Male	0.95	0.60–1.49	0.822

Severe Periodontitis			
	OR	95% CI	*p*-value
Oncologic cohort			
Control	1.0 (Ref)		
BMC	1.0	0.56–1.80	0.988
HNC	3.19	1.91–5.33	** < 0.0001**
Age (decades)			
0–49	1.0 (Ref)		
50–59	3.03	1.49–6.19	**0.002**
60–69	4.83	2.34–9.98	** < 0.0001**
>70	6.22	3.06–12.64	** < 0.0001**
Smoking			
No	1.0 (Ref)		
Yes	2.26	1.42–3.58	**0.001**
Sex			
Female	1.0 (Ref)		
Male	1.58	0.99 – 2.49	0.06

Teeth to be extracted			
	Estimate	Standard Error	*p*-value
Oncologic cohort	0.59	0.28–0.89	0.84
Age (decades)	0.47	0.26–0.68	0.06
Smoking	1.58	1.09–2.06	** < 0.0001**

In particular, smoking was associated with an OR of 3.22 (95% CI: 1.93–5.35, *p* < 0.0001), whereas HNC was associated with an OR of 2.36 (95% CI: 1.35–4.13, *p* < 0.0001). No significant differences were found between the BMC group and the control group (*p* = 0.430) for poor OH.

When the OH variable was stratified by decades, patients older than 70 years had an OR of 17.44 (95% CI: 8.78–34.66, *p* < 0.0001) for poor OH, compared with patients younger than 50 years. This risk decreased for younger patients: for patients aged 60–69 years, the OR was 5.17 (95% CI: 2.74–9.75, *p* < 0.0001); for patients aged 50–59 years, it was 4.22 (95% CI: 2.30–7.76, *p* < 0.0001).

Furthermore, a sensitivity analysis was performed to specifically investigate the relative impact of smoking and sex on the main results. The results of this analysis are depicted in supplementary materials 1, and did not show any substantial differences with the main analysis, confirming age, smoking and HNC as the main risk factors for poor OH.

**Fig. 2:**
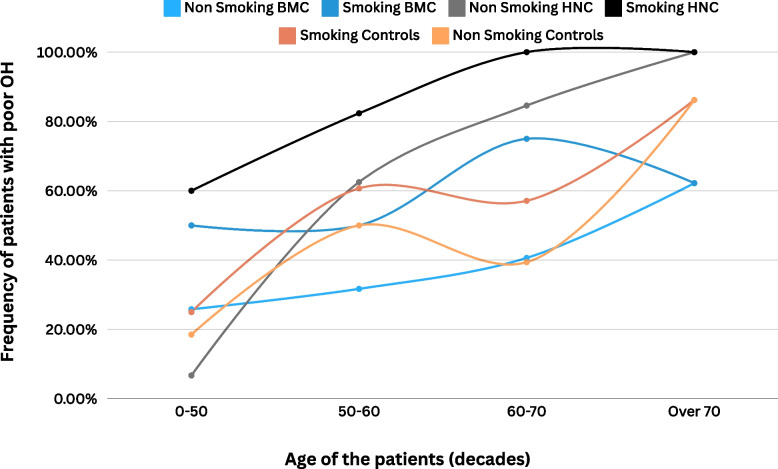
Figure 2 shows an explorative visual model, reporting the percentage of patients with poor OH for each age decade, stratified according to smoking and oncological cohort. Age significantly impacted on the OH conditions of the included, as well as HNC (black and grey curve) and smoking

Figure 2 shows an explorative visual model, reporting the percentage of patients with poor OH for each age decade, stratified according to smoking and oncological cohort.

Similarly, for the outcome “Severe Periodontitis”, age > 49 years remained a statistically significant risk factor, with ORs increasing for each decade (OR for 50–59: 3.03, 95% CI: 1.49–6.19; OR for 60–69: 4.83, 95% CI: 2.34–9.98; OR for > 70: 6.22, 95% CI: 3.06–12.64), as well as smoking habit (OR: 2.26; 95% CI: 1.42–3.58, *p* = 0.001). HNC patients showed an OR of 3.19 (95% CI: 1.91–5.33, *p* < 0.0001) when compared to control group. No differences were found between BMC patients and control group.

For the outcome DMFt ≥ 13 (i.e., severe caries experience), compared with both the HNC patients and the control patients, the BMC patients had less severe caries experience (OR: 0.51, 95% CI: 0.29–0.87, *p* = 0.013). For HNC patients, the OR was 2.0 (95% CI: 1.19–3.35, *p* = 0.008). Smoking (OR:2.72; 95% CI: 1.70–4.36, *p* < 0.0001) and age > 49 years were statistically associated also with this outcome variable (OR for 50–59: 5.4, 95% CI: 2.76–10.57; OR for 60–69: 4.96, 95% CI: 2.48–9.92; OR for > 70: 16.36, 95% CI: 8.07–33.13).

Finally, only smoking habits were associated with a greater number of TEs (estimate: 1.58, SE: 1.09–2.06, *p* < 0.0001). Age and oncologic cohort did not show any statistically significant association with this latter outcome.

## Discussion

The present case‒control study aimed to evaluate the OH status of two different cohorts of oncologic patients compared with healthy controls. The need to develop this specific comparison arose from two instances: first, the increasing incidence of MRONJ makes it necessary to perform a dental evaluation to identify any oral foci that could trigger MRONJ. Similarly, numerous studies have shown that poor OH is a risk factor for ORN. However, while there is evidence demonstrating a correlation between HNC and poor OH, evidence describing the OH of the BMC population is very scarce [7]. It was therefore hypothesized that BMC patients would have better OH status than HNC patients, both because of the absence of anatomical correlations between the oral cavity and BMC and because of the different underlying risk factors for these malignancies.

This hypothesis was confirmed by the actual findings: in the multivariate analysis, the HNC patients had an OR of 2.36 (95% CI: 1.35–4.13, *p* < 0.0001) for poor OH. Furthermore, HNC patients had the highest prevalence of severe periodontitis (82/170, 54% of the cases), with an OR of 3.19 (95% CI: 1.91–5.33, *p* < 0.0001), compared with healthy controls (24.1%). The BMC patients had a similar prevalence of periodontitis (18.3%), but the difference was not significant (*p* = 0.988).

Compared with an updated systematic review, which estimated a 23.6% prevalence of severe periodontitis [27], it may be concluded that, while BMC patients and healthy controls had periodontal conditions similar to those of the general population, HNC patients had significantly more severe periodontal conditions.

These findings are in agreement with the available literature, which highlighted higher levels of gingival inflammation and poor OH in patients with HNC than in patients with other types of cancer [28], as well as a high burden of periodontitis, as reported by Abou-Bakr et al. [29], indicating that prompt oral assessment and an effective oral hygiene management plan are needed at the time of HNC diagnosis.

The reasons for these findings may differ and might include the overlapping of several risk factors, such as smoking habits, low socioeconomic status (SES), and alcohol consumption. Smoking habits may be a possible explanation for the compromised conditions of HNC patients, being tobacco smoking one of the main risk factors for HNC and periodontitis, as previously diffusely reported [30].

Smoking has been shown to affect the vasculature, the humoral and cellular immune responses, cell signaling processes, and tissue homeostasis, thus increasing the risk for periodontitis [31]. On the other hand, tobacco smoking is responsible for the greatest burden of HNC, as highlighted in a recent study, due to its well-known carcinogenic effects, which can ultimately cause DNA damage and gene mutations [32].

Nevertheless, although smoking habit was found to be a significant risk factor for poor OH (OR: 3.22, 95% CI: 1.93–5.35, *p* < 0.0001), it should be highlighted that multivariate analysis identified HNC as an independent risk factor for poor OH, which probably requires the evaluation of different hypotheses underlying this association. For example, periodontitis contributes, throughout different pathways, to systemic inflammatory processes that may also be related to carcinogenesis [33] [34], and scattered evidence suggests that periodontitis can be an individual risk factor for oral cancer development [35].

Additionally, alcohol consumption, one of the main risk factors for HNC [36], has been associated with caries [37] and periodontitis [38]. However, in the present sample, only 4 heavy drinkers were included, and the impact of alcohol consumption to poor OH did not emerge due to its reduced prevalence. Moreover, low SES is a well-known risk factor for both HNC and dental diseases [39, 40], and people with low SES, despite reporting similar or even lower average levels of alcohol consumption, usually have worse OH conditions [41].

Unfortunately, this variable was not assessed in the present case‒control study, given that privacy concerns did not allow any investigation of the economic conditions of the included patients.

The multivariate analysis revealed a significant impact of age on the outcomes of interest, with ORs ranging from 4.22–17.44. Several papers agree with the present results, reporting worse OH in older patients, considering that age is considered a risk modifier for the onset of periodontitis [42]. Age-associated molecular alterations affect antimicrobial function in periodontitis patients, resulting in dysregulation of the inflammatory response. Thus, an age-related, increased susceptibility to periodontitis in older people is biologically plausible [43].

Of note, the mean age of onset may significantly differ between HNC patients and BMC patients. In fact, in the general population, the age of onset for HNC has been reported to be older than 75 years in 22% of patients [44], whereas the median age of onset for breast cancers (the majority of the BMC cohort) is usually 62 years [45]. However, this potential limitation was prevented, considering that the two groups were matched by age.

Furthermore, caries experience was significantly greater for HNC patients than for the control group: OR 2.0 (95% CI 1.19–3.35, *p* = 0.008) for a DMFT score ≥ 13. This finding confirms one reported in a recent paper by Jebril et al. [46], who reported an average DMFT score of 19.9 in an HNC cohort before RT.

The reasons behind this association should be investigated while considering the oral dysbiosis associated with both dental caries and oral cancers. In fact, interactions between the commensal flora and the host stimulate local mucosal and systemic immunity. Dysregulation of these complex interactions might result in the loss of antitumor effects [47]. Moreover, excessive sugar intake is an established risk factor for dental caries [48], and some papers have reported an association between malnutrition and HNC [49].

The OH status of the BMC population was similar to that of the control group, in accordance with the findings of a previous paper by Ciardo et al. [50], and the incidence of caries was lower in the BMC population than in the control group (OR: 0.51, 95% CI 0.29–0.97, *p* = 0.013). Regarding this rather surprising result, it is possible to assume that, as the control group patients were selected from an outpatient hospital population, they had reduced financial resources, which might have limited their access to preventive dentistry protocols in the past. Instead, it is possible to assume that BMC patients had undergone preventive dentistry protocols, which could explain the reduced prevalence of caries. However, further studies, better controlled for socio-economic conditions of the included patients, might help to validate this finding.

In any case, there was a tendency for BMC patients to have even better OH than controls did, although the difference was not statistically significant. This finding might also be explained by the different distributions of smokers between the two groups. Having such a good OH status is certainly comforting, since there is evidence that poor OH is associated with a greater risk of MRONJ [51]. Nonetheless, 44.7% (76/170) of BMC patients still had poor OH, which was mostly determined by a history of periodontitis. This resulted in the need to perform 0.9 TEs per patient, an event that slows the patient’s access to AR therapy. A limited number of cohort studies have investigated the association between periodontal disease and breast cancer. Soder et al. [52], as well as Freudenheim et al. [53], identified an association between periodontitis and postmenopausal breast cancer. Conversely, our findings confirm the results of two more recent cohort studies, which failed to identify a clear association between periodontitis and breast cancer [54, 55]. However, no definite conclusions can be drawn, especially considering that our study was not longitudinal and did not aim to investigate any causal relationship between BMC and periodontitis. Confirmation of the association of breast cancer with periodontitis requires further definitive studies [56]. In addition, given its chronic nature, untreated periodontitis may increase the risk of tooth loss, which, in such fragile patients, may lead to discontinuation of AR therapy or, in the worst cases, trigger MRONJ [57, 58]. Furthermore, the most recent evidence suggests that a periodontal infectious process, even without any dentoalveolar surgical interventions, can trigger MRONJ [59, 60]. These considerations reinforce the indication for secondary prevention of dental and periodontal diseases, as well as, of course, emphasize the need for better primary prevention protocols.

In conclusion, the number of required TEs did not differ between the oncologic groups (*p* = 0.84). This is probably because this variable is not necessarily the expression of poor OH: patients who are already completely or partially edentulous may have no need for TE, but they would suffer from poor OH.

One of the main strengths of this study is that, to the best of the authors’ knowledge, this is the first paper investigating OH in a BMC population waiting for the beginning of AR drug treatment. This is probably because interest in the OH of these patients is a relatively recent trend that is likely to increase in the coming years, considering its impact on the risk of MRONJ [17].

The present study also has the following limitations: the reason for tooth loss (caries or periodontitis) was investigated retrospectively on the basis of anamnesis. This may have led to an overestimation of the prevalence of one disease over the other. Consequently, the results of the multivariate statistical analysis must be considered with caution, particularly with respect to caries experience (i.e., BMC cannot be considered a protective factor from caries), as discussed above. However, the use of the OH parameter limits the extent of this potential bias since it integrates the two main variables of interest. Additionally, this study has a monocentric design, which may have limited the reliability of our results on a larger scale. Furthermore, the oncologic cohorts were not matched according to sex and smoking habits. This was inevitable, given that the vast majority of patients in the BMC cohort were breast cancer patients and that HNC is more common in male subjects. In addition, smoking is the main risk factor for the development of HNC; therefore, the number of smokers was unavoidably greater in this cohort. However, multivariate logistic regression excluded a statistically significant impact of sex on the outcomes of interest, and while smoking remained a significant risk factor for poor OH, the HNC oncologic cohort was still found to be an independent risk factor, thus confirming the study hypothesis.

Another potential limitation of this study is that the data were obtained from a secondary analysis of patients initially enrolled in different clinical studies. This entails an increased risk of selection bias. However, only 26 patients out of a final sample of 510 (8 belonging to the HNC group and 18 to the control group) were excluded to ensure correct age matching. In all other cases, the inclusion and exclusion criteria were strictly applied, and the selection bias was consequently limited. Furthermore, the inclusion of patients in prospective studies allowed a detailed data collection, eliminating the presence of missing data, another bias common to retrospective studies.

## Conclusions

HNC patients exhibit significantly poorer oral health than BMC patients and healthy controls. This study confirms the associations between caries and periodontitis and head and neck cancer.The present findings highlight the need for enhanced dental care in oncologic management, confirming how prevention protocols are needed to reduce the burden of periodontitis and avoid the need for tooth extraction, which may, for both cohorts, slow access to oncologic therapy.

## Supplementary Information

Supplementary Material 1.

## Data Availability

The datasets generated and/or analyzed during the current study are available from the corresponding author on reasonable request.

## Abbreviations

BMC: Bone Metastatic Cancer
HNC: Head and Neck Cancer
OH: Oral Health
RT: Radiotherapy
AR: Antiresorptive (drugs)
DMFT: Decayed, Missing, and Filled Teeth (index)
TE: Tooth Extraction
OR: Odds Ratio
CI: Confidence Interval
MRONJ: Medication-Related Osteonecrosis of the Jaw
CTIBL: Cancer Treatment-Induced Bone Loss
ORN: Osteoradionecrosis
OPT: Orthopantomograph
PPD: Probing Pocket Depth
CAL: Linical Attachment Loss
SD: Standard Deviation
IQR: Interquartile Range
SPSS: Statistical Package for the Social Sciences
SES: Socioeconomic Status
ASA: American Society of Anesthesiologists
