# Supplementary material for: Differences in oral health status between cancer patients: a case–control observational study
Source: BMC Oral Health. 2025 Dec 1;25:1862. doi: 10.1186/s12903-025-06963-7 (PMC12670815; doi:10.1186/s12903-025-06963-7)
Supplement: Supplementary file 1 — Supplementary Material 1. [file 12903_2025_6963_MOESM1_ESM.docx]

| Poor OH | | | |
| --- | --- | --- | --- |
| Group A: Male subjects (n of patients: 245) | | | |
| *Predictors* | OR | 95% CI | p-value |
| Oncologic cohort |  |  |  |
| Control | 1.0 (Ref) |  |  |
| BMC | 1.30 | 0.46 – 3.69 | 0.618 |
| HNC | 6.03 | 1.93 – 18.86 | **0.002** |
| Age (decades) |  |  |  |
| 0-49 | 1.0 (Ref) |  |  |
| 50-59 | 4.91 | 2.04 – 11.77 | **<0.0001** |
| 60-69 | 6.19 | 2.53 – 15.14 | **<0.0001** |
| >70 | 24.96 | 8.70 – 71.55 | **<0.0001** |
| Smoking |  |  |  |
| No | 1.0 (Ref) |  |  |
| Yes | 3.15 | 1.49 – 6.65 | **0.003** |
| Group B: Female subjects (n of patients: 265) | | | |
|  | OR | 95% CI | p-value |
| Oncologic cohort |  |  |  |
| Control | 1.0 (Ref) |  |  |
| BMC | 1.55 | 0.81 – 2.99 | 0.188 |
| HNC | 3.73 | 1.89 – 7.39 | **<0.0001** |
| Age (decades) |  |  |  |
| 0-49 | 1.0 (Ref) |  |  |
| 50-59 | 3.82 | 1.72 – 8.48 | **0.001** |
| 60-69 | 4.09 | 1.74 – 9.61 | **0.001** |
| >70 | 11.49 | 4.72 – 27.96 | **<0.0001** |
| Smoking |  |  |  |
| No | 1.0 (Ref) |  |  |
| Yes | 3.26 | 1.59 – 6.65 | **<0.0001** |
| Group C: Smokers (n of patients: 177) | | | |
|  | OR | 95% CI | p-value |
| Oncologic cohort |  |  |  |
| Control | 1.0 (Ref) |  |  |
| BMC | 1.71 | 0.49 – 5.92 | 0.396 |
| HNC | 4.14 | 1.28 – 13.35 | **0.02** |
| Age (decades) |  |  |  |
| 0-49 | 1.0 (Ref) |  |  |
| 50-59 | 4.71 | 1.71 – 12.92 | **0.003** |
| 60-69 | 9.33 | 2.67 – 32.63 | **<0.0001** |
| >70 | 17.57 | 4.28 – 72.18 | **<0.0001** |
| Sex |  |  |  |
| Female | 1.0 (Ref) |  |  |
| Male | 1.63 | 0.64 – 4.13 | 0.303 |
| Group D: Smokers (n of patients: 333) | | | |
|  | OR | 95% CI | p-value |
| Oncologic cohort |  |  |  |
| Control | 1.0 (Ref) |  |  |
| BMC | 1.60 | 0.94 – 2.74 | 0.08 |
| HNC | 3.35 | 1.68 – 6.71 | **0.001** |
| Age (decades) |  |  |  |
| 0-49 | 1.0 (Ref) |  |  |
| 50-59 | 3.62 | 1.74 – 7.55 | **0.001** |
| 60-69 | 4.14 | 1.95 – 8.79 | **<0.0001** |
| >70 | 16.54 | 7.52 – 36.39 | **<0.0001** |
| Sex |  |  |  |
| Female | 1.0 (Ref) |  |  |
| Male | 1.62 | 0.93 – 2.82 | 0.09 |

**Supplementary Materials 1.** Sensitivity analysis for poor Oral Health. The analysis was performed to highlight any differences in the main results when considering only male (group A) or female subjects (group B), or only smokers (Group C) and non-smokers (Group D).
